# Superfine Grinding for Edible Powders: Mechanisms, Quality Regulation, Limitations, and Synergistic Strategies

**DOI:** 10.3390/foods15122050

**Published:** 2026-06-06

**Authors:** Jiangfeng Yang, Yimeng Ren, Bengkang Xie, Chu Wan, Shuquan Xin, Kai Song

**Affiliations:** 1 School of Life Science, Changchun Normal University, Changchun 130032, China; abcyifdd@163.com (J.Y.); rym351528@163.com (Y.R.); kkangyyds@163.com (B.X.); wanchuccsf@163.com (C.W.); 2 Institute of Innovation Science and Technology, Changchun Normal University, Changchun 130032, China

**Keywords:** mechanical micronization, cell-wall disruption, bioaccessibility, particle agglomeration, mechanochemistry, synergistic processing

## Abstract

Edible powders are important food ingredients, and their quality strongly affects processability, stability, and nutrient delivery. Compared with conventional grinding, superfine grinding enables particle-size reduction to the micron or submicron scale and has shown considerable potential for improving the physicochemical and functional properties of food powders. This review summarizes five representative superfine grinding technologies and discusses how different mechanical force fields regulate powder quality through changes in particle size, specific surface area, cell-wall integrity, and macromolecular structure. Current evidence indicates that superfine grinding can improve hydration behavior, dissolution, the release of bioactive compounds, antioxidant activity, and in vitro bioaccessibility, but these effects are highly dependent on raw-material characteristics and processing conditions. At the same time, excessive micronization may induce particle agglomeration, thermal degradation of sensitive components, sensory deterioration, high energy consumption, and potential safety concerns related to ultrafine particles. Therefore, the performance of a single grinding technology is often constrained by intrinsic physicochemical and engineering limitations. Recent studies suggest that combining superfine grinding with pretreatment, interfacial stabilization, or encapsulation strategies can improve powder stability and functionality more effectively than grinding alone. Future research should focus on standardized evaluation systems, mechanistic clarification across food matrices, and integrated process design for industrial application.

## 1. Introduction

Edible powders are finely processed solid particulate ingredients derived from plant, animal, fungal, or microbial sources. They are widely used in the modern food industry not only as nutritional and bioactive carriers, but also as functional ingredients that enhance sensory attributes (e.g., flavor, color, and texture), serve decorative purposes (e.g., powdered toppings, dustings, and coatings), and act as technological aids in formulation (e.g., thickeners, dispersants, and free-flow agents) [1,2]. Their applications span solid beverages, bakery products, dairy products, confectionery, meal-replacement foods, seasonings, and a wide range of functional and medicinal-and-edible homologous products. The physicochemical properties of powders directly determine the sensory quality, nutritional nutrient retention [3], and physiological activity of the final products. Comminution is a fundamental step in preparing edible powders. As a core technological operation in this process [4], the manner of applying force and the mechanical intensity during comminution determine the final particle-size distribution and strongly influence the stability of thermosensitive compounds and the targeted release efficiency of functional components [5]. Therefore, the development of efficient and precise comminution processing technologies has long been a research focus in this field [6,7].

Although conventional mechanical crushing uses simple equipment and incurs low cost, it typically yields coarse particles larger than 100 μm and suffers from a broad particle-size distribution and poor uniformity [8]. Moreover, the intense frictional heat generated during coarse processing likely causes irreversible degradation of heat-sensitive nutrients and bioactive compounds. The resulting powder often exhibits poor solubility and dispersibility [9], which limits bioavailability and makes it difficult to meet modern food-industry requirements for producing high-value ingredients. Superfine grinding (also referred to as ultrafine grinding or micronization) is defined in this review as a mechanical comminution process that reduces solid food materials to a median particle size (D50) typically below 25 μm, and in some cases to the submicron or nanoscale (≥100 nm), by applying high-intensity mechanical force fields such as high-speed impact, airflow shear, media abrasion, hydrodynamic cavitation, or rotor–stator shear [1,6,8]. Compared with conventional grinding (D50 > 100 μm), superfine grinding generates particles with a markedly larger specific surface area, more uniform size distribution, and substantially altered surface thermodynamic states, which collectively underpin its functional advantages in food powder processing. This extreme particle-size reduction and homogenization markedly improve powder dispersion, dissolution, and bioavailability while preserving nutrients and active compounds within natural matrices. In recent years, the technique has gained considerable attention in food processing [10].

Several reviews have addressed the application of ultrafine grinding to food powder processing. For example, Gao [6] provided a preliminary summary of mainstream ultrafine grinding processes and their effects on powder physicochemical properties, while Duguma et al. evaluated the technology’s potential for improving powder quality control. However, existing research remains insufficient in three dimensions. First, most studies evaluate individual processes in isolation and do not perform cross-technology comparisons across heterogeneous feedstocks, which hinders the formulation of matching criteria between raw-material characteristics and technology selection. Second, single refinement technologies show serious physicochemical limitations in practice—such as powder agglomeration [11], high energy consumption [12], and poor adaptability to special materials [13]—and the internal causes and interactions among these limitations have not been systematically explained. Finally, although combining multiple technologies has emerged as the primary route to overcome the limits of any single approach, a systematic review of their mechanisms of action, applicable boundaries, and targeted limitations is lacking [14,15]. This gap restricts industrial translation and prevents standardized evaluation.

Beyond the three gaps identified above, the construction of safety supervision and evaluation standards for superfine grinding has also received only limited attention in the existing literature.

To address these gaps, the present review is organized along a progressive logical line that moves from technical characteristics through physicochemical regulation and limiting causes, culminating in synergistic breakthroughs. Specifically, we (i) systematically compare the mechanical action mechanisms and applicability of five representative technologies—jet mill, ball mill, vibration mill, high-pressure homogenizer, and colloid mill; (ii) analyze how detailed processing conditions govern key physicochemical indicators, including particle size, hydration kinetics, dissolution of active ingredients, antioxidant capacity, and dietary fiber structure; (iii) emphasize the common limitations of each single technology and propose targeted synergistic strategies; and (iv) provide an overview of current safety challenges and the corresponding needs for standard development.

**Figure 1:**
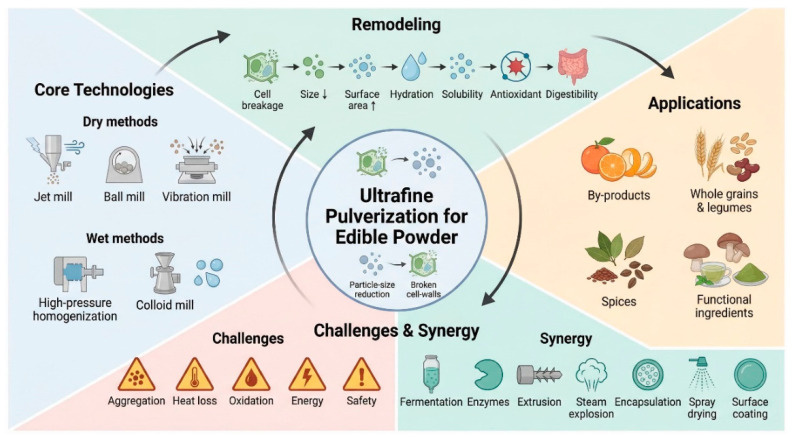
Schematic framework of the core action mechanisms, multidimensional quality-regulation network, and synergistic optimization strategies of superfine grinding technology in edible-powder preparation.

On this basis, the central viewpoint of this review is that the regulation of powder quality by superfine grinding exhibits pronounced raw-material specificity and strong process dependence; the intrinsic limitations of any single technology cannot be eliminated by parameter tuning alone, and cross-integration of multiple technologies therefore represents a necessary route toward food powders with both high functionality and high stability. The absence of a systematic evaluation framework remains a major obstacle to large-scale implementation. The overall framework of this review is illustrated in Figure 1.

Literature search strategy. This review was compiled through a systematic literature search performed in the Web of Science Core Collection, Scopus, PubMed, and ScienceDirect databases. The search period covered publications from January 1997 to January 2026. The following keywords and Boolean combinations were used: (‘superfine grinding’ OR ‘ultrafine grinding’ OR ‘micronization’ OR ‘jet milling’ OR ‘ball milling’ OR ‘high-pressure homogenization’ OR ‘colloid mill’ OR ‘vibration mill’) AND (‘edible powder’ OR ‘food powder’ OR ‘physicochemical properties’ OR ‘bioactive compounds’ OR ‘dietary fiber’ OR ‘particle size’). Only peer-reviewed articles published in English were included. The retrieved references were screened by title, abstract, and full text to ensure relevance to food-grade superfine grinding.

## 2. Major Superfine Grinding Technologies

**Figure 2:**
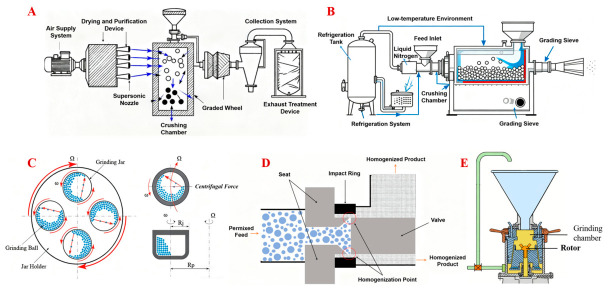
Schematic working principles of mainstream superfine grinding technologies: (**A**) jet mill; (**B**) cryogenic vibration grinding integrated with a cooling module; (**C**) mechanical mechanism of ball milling, adapted from Gao et al. [6]; (**D**) hydrodynamic cavitation chamber of high-pressure homogenization, adapted from Gao et al. [6]; (**E**) colloid mill.

According to differences in dispersion media and rheological environment, superfine grinding technologies can be broadly divided into dry and wet systems [6,7]. Dry grinding uses gases or solid media as energy-transfer carriers, and representative devices include ball mills, jet mills, and vibration mills. Wet grinding, by contrast, employs a liquid phase as the dispersion medium and mainly involves equipment such as colloid mills and high-pressure homogenizers. These two technological systems differ markedly in material-form requirements, mechanical force fields, and process integration. Their major technologies and operating principles are illustrated in Figure 2. Regarding the characteristics and applications of the devices, please see Table 1 for details.

### 2.1. Jet Mill

A jet mill, also known as a fluid-energy mill, is a dry, media-free superfine grinding device that uses high-velocity gas streams as the kinetic-energy carrier. Its core components are the nozzle and grinding chamber, and the process is governed by a purely physical autogenous grinding mechanism [16,17]. As shown in Figure 2A, dried and filtered compressed gas is accelerated through the nozzle into a supersonic impact stream, thereby generating a high-velocity flow field in the grinding chamber. Once introduced into this field, particles are micronized through intense interparticle collision, wall impact, and gas-stream-induced shear friction [18]. An internal classifier wheel retains coarse particles for recirculated grinding, whereas qualified fine particles exit with the gas stream. Fluidized-bed, flat spiral, and opposed-jet configurations are currently the most widely used types in edible-powder processing [19].

In food engineering, jet milling offers several distinctive advantages. First, because no metallic grinding media are involved, inorganic contamination is fundamentally avoided, yielding powders of high purity and highly uniform particle-size distribution [20]. Second, the adiabatic expansion of compressed gas at the nozzle absorbs large amounts of heat from the system, resulting in minimal temperature rise within the grinding chamber and providing intrinsic protection against degradation of thermosensitive raw materials [21]. Studies have shown that jet milling can simultaneously reduce cereal endosperm and bran to uniformly micronized dimensions, thereby overcoming the large bran particle size and broad distribution characteristic of conventional grinding; moreover, the resulting whole-grain flour exhibits significantly higher retention of dietary fiber and polyphenols than flour produced by conventional mechanical grinding [22]. However, jet milling also shows clear limitations in scale-up. The need for compressed gas leads to high overall energy consumption and therefore high unit production cost [6]. System throughput is constrained by gas-flow behavior and classification efficiency [12]. In addition, jet milling imposes strict requirements on the mechanical properties of the substrate and is mainly suitable for dry, brittle solids with low moisture content [23].

### 2.2. Ball Mill

Ball milling reduces materials primarily through mechanical stresses—impact and shear—generated by grinding media such as steel or ceramic balls during motion [24]. During operation, the feed material and grinding media are loaded into the milling chamber at a defined filling ratio. Under the combined action of centrifugal force and gravity, the media exert frequent sliding, rolling, and compressive stresses on particles (Figure 2C) [6], ultimately causing disruption of the microstructure [10]. Depending on the dispersion environment, ball milling can be operated in dry or wet mode. In wet ball milling, the liquid medium effectively suppresses the spontaneous agglomeration of very small particles caused by high surface energy [25], thereby conferring better dispersion stability on the resulting powder [7]. Consequently, wet ball milling has been more widely used in the preparation of fruit, vegetable, and cereal powders.

The key advantage of ball milling lies in its broad material adaptability. It can effectively process fibrous and tenacious substrates and also shows high tolerance toward high-moisture raw materials. In addition, its robust equipment structure and relatively low operation and maintenance cost make it suitable for continuous production of low-value food materials, while the enclosed grinding chamber effectively limits dust emission. Experimental evidence indicates that wet ball milling can reduce corn starch with a moisture content as high as 30% to the submicrometer scale and simultaneously disrupt crystalline microdomains within starch granules, thereby markedly improving hydration, solubility, and pasting rheology [26]. However, its intrinsic drawbacks are equally evident. Long-term friction between the grinding media and the chamber wall can introduce trace metallic or ceramic debris into the powder [27], thereby creating a risk of media-derived contamination [28]. Extended mechanical friction also causes pronounced temperature rise, making it difficult to protect thermolabile constituents. Moreover, grinding efficiency is subject to nonlinear interactions among multiple factors, including media ratio and rotational speed, often resulting in broad particle-size distribution and limited controllability of uniformity [7]. The system is also associated with high noise levels and considerable mechanical energy consumption [6].

### 2.3. Vibration Mill

A vibration mill operates by using an eccentric excitation device to drive the grinding vessel into high-frequency periodic vibration [29], thereby converting vibrational energy into intense impact, shear, and compressive action of the grinding media on the material. Unlike the low-speed rotational motion of a ball mill, particles in a vibration mill are subjected to high-intensity microscopic stresses between the media—ceramic balls, metal balls, or rods—and the vessel wall [30]. Owing to its higher media filling ratio and greater instantaneous impact frequency [31], the fracture efficiency of a vibration mill is markedly higher than that of conventional ball milling.

Vibration milling is particularly effective for disrupting plant-fiber-rich matrices. Its high-frequency stress field efficiently breaks down the cell-wall skeletons of whole grains, fruits, vegetables, and mushrooms, thereby promoting the targeted release of endogenous macromolecules, such as dietary fiber and polysaccharides, as well as secondary metabolites, such as polyphenols; in addition, the resulting powders generally exhibit a more concentrated particle-size distribution. Nevertheless, several engineering limitations arise when this equipment is used for functional ingredient production. High-frequency vibration entails substantial noise-control and maintenance costs. Rapid temperature rise caused by intensive mechanical friction readily induces oxidation or volatilization of thermolabile constituents such as aroma compounds and phenolics. Media-wear contamination, similar to that observed in ball milling, also remains a concern. To address these thermodynamic drawbacks, current equipment designs increasingly integrate cryogenic modules, for example liquid-nitrogen cooling systems, to establish low-temperature vibration-grinding platforms (Figure 2B), thereby enabling high-value fine processing of low-melting, highly thermosensitive, and volatile materials.

### 2.4. High-Pressure Homogenization

High-pressure homogenization (HPH) is a wet superfine-size reduction process that relies entirely on fluid-dynamic effects, including high-pressure shear, ultrafast impact, and cavitation [32]. After pretreatment into a uniform suspension, the material is pressurized by a high-pressure pump and forced through the narrow gap of the homogenizing valve. The sharp increase in flow velocity generates intense shear, while the rapid pressure drop triggers the collapse of cavitation bubbles; the combined action of these physical fields fragments suspended particles (Figure 2D) [6,33].

HPH offers three major advantages in edible-powder processing. First, because the mechanism is purely fluid-dynamic and does not involve solid grinding media, metallic contamination is fundamentally avoided, conferring a high level of process safety. Second, the residence time under stress within the homogenizing valve is extremely short, which effectively prevents system overheating and provides superior protection for thermolabile factors such as vitamins, polyphenols, natural pigments, and bioactive peptides compared with conventional mechanical grinding. Third, the resulting particles are highly uniform in size, which substantially enhances dispersion stability in liquid systems. The main limitations of HPH stem from the fact that the feed must be a low-viscosity liquid or slurry and therefore requires both upstream slurry preparation and downstream drying, which lengthens the powder-production process and increases both energy demand and operational complexity. In addition, the highly precise structure and key components are prone to wear, leading to relatively high operation and maintenance cost. HPH also shows poor adaptability to highly fibrous or very hard solid particles, which can readily reduce homogenization efficiency or cause line blockage. Furthermore, although the fluid forces generated by HPH are generally insufficient to disrupt small molecules such as amino acids and pigments, they may induce varying degrees of spatial rearrangement in larger biomolecules such as proteins and enzymes.

### 2.5. Colloid Mill

A colloid mill applies intense hydrodynamic shear, interfacial friction, high-frequency impact, and turbulence to flowing wet materials through the very narrow gap between a rotor and a stator rotating at high relative speed, thereby rapidly disintegrating and refining their microstructure (Figure 2E) [34].

The most notable advantage of a colloid mill is its broad applicability to diverse raw materials. In addition to low-viscosity feeds, it can effectively process semisolid fluids with high viscosity and high solids content, such as pastes and slurries [35]. With high refinement efficiency and relatively uniform particle size, the technology can also reduce material agglomeration effectively, making it an ideal pretreatment unit in wet powder-manufacturing processes for generating stable and homogeneous feed liquids prior to spray drying or freeze drying. Its strong shear field can further disrupt plant cell walls and facilitate the release of functional constituents. Because the equipment is structurally simple, compact, and easy to maintain, it is widely used in small and medium-sized food enterprises. However, the technological ceiling of colloid milling is relatively clear. Owing to the physical limits imposed by machining precision, particle size after grinding generally remains in the micrometer range and cannot be reduced to the nanoscale. Continuous high-speed shear friction between the rotor and stator also introduces heat into the slurry; without efficient cooling, thermolabile compounds may undergo degradation or inactivation. In addition, a downstream drying step is still required to obtain the final powder product.

**Table 1:** Characteristics and applications of five representative superfine grinding devices.

Technology	Grinding Medium	Applicable Material State	Particle-Size Range	Protection of Thermolabile Components	Contamination Risk	Typical Raw Materials	Reference
Jet mill	High-velocity gas (no solid medium)	Dry, brittle solids	1–20 μm	Excellent (adiabatic expansion)	None	Cereal flours, spices, mushroom powders	[18]
Ball mill	Steel balls/ceramic balls	Dry or wet solids	1–100 μm	Poor (frictional heating)	Medium-high	Starch, fruit and vegetable powders, traditional herbal materials	[6]
Vibration mill	Ceramic balls/rods	Dry or wet solids	1–50 μm	Poor (rapid heating; improvable under cryogenic conditions)	Medium	Fibrous materials, grains, mushrooms	[29]
High-pressure homogenization	None (fluid dynamics)	Liquids or slurries	0.1–10 μm	Good (instantaneous action)	None	Emulsions, herbal extracts, proteins	[33]
Colloid mill	Rotor–stator (fluid shear)	Wet, high-viscosity slurries	1–50 μm	Moderate (continuous shear causes some heating)	Low	Fruit and vegetable purees, cereal emulsions, spices	[34]

## 3. Effects of Superfine Grinding on the Physicochemical Properties of Edible Powders

When superfine grinding alters the physicochemical properties of powders, these changes do not occur independently but unfold along a strict causal sequence: particle-size reduction and specific-surface-area expansion → disruption of thermodynamic equilibrium through increased surface free energy → cell-wall disintegration and macromolecular conformational modification → shifts in hydration behavior and active-compound release → enhancement of antioxidant potential and bioavailability. This physicochemical evolution is, however, strongly material-specific and technology-dependent. The following subsections examine in depth the mechanisms by which superfine comminution reconstructs powder quality, with reference to representative experimental evidence.

### 3.1. Particle-Size Distribution, Microstructure, and Surface Thermodynamics

**Figure 3:**
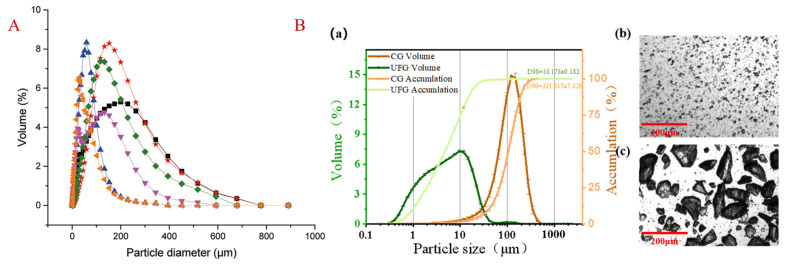
Effects of superfine grinding on physicochemical properties of edible powders: (**A**) particle-size distribution curves of hawthorn fruit powder prepared by different methods, Hot-air-dried mortar ground powder (HGP) (black, ■); hot-air-dried shear pulverized powder (HSP) (red, ★); hot-air-dried jet milled powder (HJP) (blue, ▲); freeze-dried mortar ground powder (FGP) (pink, ▼); freeze-dried shear pulverized powder (FSP) (green, ◆); freeze-dried jet milled powder (FJP) (orange, ◀). adapted from Liu et al. [41]; (**B**) particle-size distribution (**a**) and micrographs (**b**,**c**) of *Tremella fuciformis* powder prepared by superfine grinding, adapted from Zhang et al. [42].

The geometric reduction in particle size is the most direct physical consequence of superfine grinding and the thermodynamic origin of many subsequent property changes [36,37]. Studies consistently show that, with increasing grinding time, particle size generally follows a nonlinear pattern characterized by an initial rapid decline followed by a plateau. For example, under vibration milling equipped with an integrated cooling system, the median diameter of moso bamboo shoot powder decreased to 18.67 μm and 10.35 μm after 30 and 60 min of treatment, respectively [38]. Similarly efficient size reduction has been reported for buckwheat flour [39] and soybean residue [40], both showing particle-size decreases exceeding 79%. Under different mechanical force fields, hawthorn fruit powder [41] and *Tremella fuciformis* powder [42] displayed distinct particle-size distribution profiles (Figure 3(A,Ba)), directly reflecting the varying capacity of different technologies to regulate size uniformity.

The trajectories of morphological and structural change depend to a large extent on the original internal organization of the raw material. After grinding, Tremella powder [42] exhibited a typical transformation from polyhedral particles to irregular fragments (Figure 3(Bb,Bc)). In plant tissues, Gan [43] observed that the originally compact cell-wall network of mulberry leaf powder was completely disrupted after superfine grinding, yielding uniform microfragments with rough surfaces. Proteinaceous powders, exemplified by glutinous rice protein [44], likewise changed from dense and smooth surfaces to loose and rough structures.

As particle size decreases, the specific surface area increases nonlinearly, and the surface-energy state is consequently redefined. In potato starch [45], treatment at 3000 r/min increased the specific surface area to 2.42 times that of the native powder. When particle size reaches the submicrometer range, the fraction of surface molecules rises markedly, exposing large numbers of active sites such as hydroxyl and carboxyl groups and significantly increasing surface free energy. This high-energy state renders particles thermodynamically unstable [46], making them highly prone to spontaneous aggregation via van der Waals interactions and hydrogen bonding, thereby forming secondary agglomerates [47]. Such aggregation not only deteriorates powder flowability but also constitutes the central physical contradiction in maintaining stability during micronization.

### 3.2. Hydration, Dissolution, and Targeted Release of Bioactive Compounds

Superfine grinding systematically regulates water-holding capacity (WHC), oil-holding capacity (OHC), and water solubility index (WSI) by disrupting cell walls, enlarging the solid–liquid interfacial area, and inducing partial transformation of constituents. Hydration behavior exhibits a clear threshold effect. At the initial stage of grinding, disruption of cell-wall and fiber structures exposes a large number of endogenous hydrophilic groups, including hydroxyl and carboxyl groups. The increase in water-binding sites consequently raises WHC, as demonstrated in *lentinus edodes* mushroom powder [48], apple pomace powder [49], Tremella powder [42], and pumpkin seed powder [50]. As micronization progresses, the increasingly loose particle structure enhances adsorption of lipid molecules, thereby increasing OHC [51]. In insoluble dietary fiber from Polygonatum, for instance, moderate ball milling simultaneously increased WHC, OHC, and swelling capacity to peak values of 5.12 g/g, 2.83 g/g, and 3.83 mL/g, respectively [52].

The increase in water solubility arises from two major mechanisms. First, mechanical force disrupts cell walls and releases intracellular starch and proteins. Second, mechanochemical action can degrade or transform insoluble fiber, thereby generating soluble dietary fiber [53]. In addition, particle-size reduction accelerates water uptake and dissolution kinetics. It should be noted, however, that excessive grinding may promote particle clustering; once such agglomeration reduces the effective dispersible area, the increase in WSI may level off or even decline.

Complete disruption of the cell wall is the prerequisite for the targeted release of active constituents. Plant bioactives are often entrapped within fibrous structural frameworks, and the intense mechanical force generated during superfine grinding breaks these barriers, thereby establishing efficient pathways for mass transfer between intracellular components and the external solvent.

**Figure 4:**
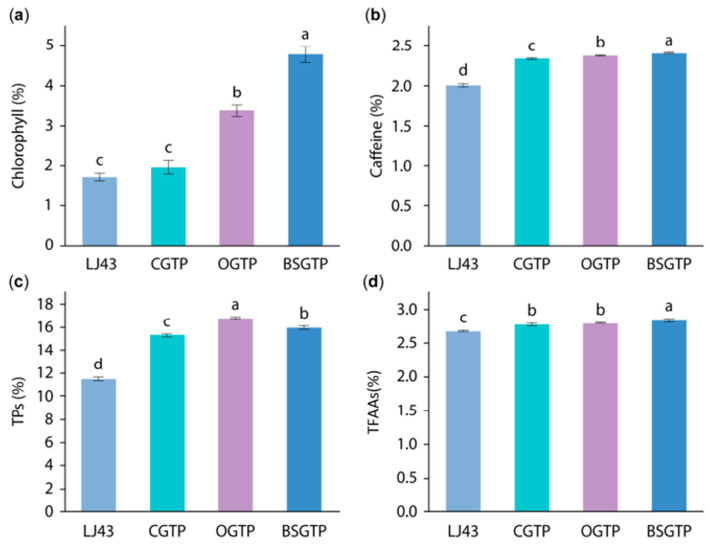
Major component contents in four groups of SGTP samples (*p* < 0.05): (**a**) chlorophyll; (**b**) caffeine; (**c**) TPs; (**d**) TFAAs. Values in a single figure with different letters are significantly different. Adapted from Zhou et al. [55].

For polyphenols and flavonoids stored in vacuoles, cell-wall disruption directly leads to rupture of the vacuolar membrane. After superfine treatment, the release rate of total polyphenols from hawthorn powder [41] increased from 35.7% to 82.3%, while flavonoid release increased 2.9-fold. In *Lycium ruthenicum* murray powder [54], the extractable contents of anthocyanins and polysaccharides increased by 72.3%. Superfine grinding can further raise the cell-wall breakage rate to 93% while reducing particle size to the micrometer scale, thereby effectively improving the extraction efficiency of major constituents. For example, in the preparation of superfine green tea powder (SGTP) by ball milling, an AHP–fuzzy evaluation model based on chlorophyll, caffeine, tea polyphenols (TPs), and free amino acids (TFAAs) was used to optimize the process. Compared with conventionally squeezed green tea powder, the optimized product, with a particle size of 4–44 μm, showed superior contents and release performance of major components (Figure 4) [55].

The release of dietary fiber and proteins is accompanied by structural transformation. Under shear stress, insoluble fiber changes from tightly bundled structures to loose fibrillar forms [56], which significantly increases the soluble fraction in mulberry leaf powder [43] and Qingke barley flour [57]. In soy protein isolate [58], mechanically induced conformational rearrangement exposes additional polar groups and thereby alters the adsorption kinetics of flavor compounds such as aldehydes and alcohols.

### 3.3. Antioxidant Activity, Bioaccessibility, and the “Benefit–Shortcoming” Trade-Off

As the release of active compounds increases, antioxidant activity is correspondingly enhanced. Greater exposure of reducible substances, particularly phenolics, provides more reactive sites for free-radical quenching, leading to simultaneous increases in DPPH (2,2-diphenyl-1-picrylhydrazyl) and ABTS (2,2′-azino-bis(3-ethylbenzothiazoline-6-sulfonic acid)) scavenging capacity as well as ferric reducing antioxidant power (FRAP).

While superfine grinding markedly enhances antioxidant activity through increased exposure of reducible substances, it is important to recognize that this benefit operates within a narrow processing window. On the positive side, *Panax notoginseng* powder [59] reached its maximum DPPH scavenging capacity at the M400 fineness, and bitter melon powder [60] achieved a 41.24% ABTS scavenging rate at 25 μm. However, on the negative side, excessive micronization simultaneously exposes labile phenolic hydroxyl groups to oxygen and frictional heat, thereby accelerating their oxidative degradation. In terms of chemical stability, the exposure of highly active surfaces increases the opportunity for functional groups to contact oxygen, thereby markedly accelerating oxidation of labile components such as unsaturated fatty acids and directly shortening product shelf life [as further detailed in Section 5.2]. For example, in *Citrus reticulata* powder [61], prolonged micronization (120 min) caused significant losses of ferulic acid, synephrine, hesperidin, and naringin; in mint powder [62], menthol content declined sharply with increasing rotational speed. Therefore, antioxidant activity as a function of grinding intensity typically follows a non-monotonic “rise-then-fall” pattern, and the optimal fineness must be experimentally identified for each matrix.

In addition, cryogenic media or active cooling strategies are essential for processing thermosensitive materials. The combined effects of structural disruption and molecular modification also improve bioavailability and bioaccessibility. Unfolding of the protein tertiary structure exposes enzymatic hydrolysis sites, destruction of starch crystallinity removes steric hindrance, and extremely small particle size facilitates penetration of digestive fluids. In vitro digestion models demonstrated that low-temperature micronization increased the intestinal bioaccessibility of polysaccharides and triterpenes in *Ganoderma lucidum* spore powder [63] from 29.5% and 5.4% to 72.5% and 32.9%, respectively. Likewise, the gastrointestinal release flux of ginsenoside Rg1 in red ginseng powder [64] increased from 1013 mAU·s to 1313 mAU·s.

### 3.4. Macromolecular Restructuring of Dietary Fiber, Starch, and Proteins

Superfine grinding induces decomposition and morphological transformation of macromolecules, thereby substantially altering the flow behavior and functional performance of dietary fiber, starch and proteins. Mechanical force can cleave certain linkages along cellulose chains, increasing the proportion of soluble fiber in materials such as Polygonatum [52] and Ophiopogon japonicus [65]. Meanwhile, exposure of polar sites on fiber surfaces strengthens complexation with bile salts. Accordingly, superfine treatment significantly enhanced bile-salt adsorption in buckwheat bran [66], hawthorn [67], and oat bran [68], providing a mechanistic basis for the improved lipid-lowering potential of plant matrices after micronization.

At the starch level, mechanical impact creates abundant physical defects on granule surfaces, leading to a sharp decline in crystallinity and shifts in gelatinization thermodynamics. After micronization, *Dioscorea opposita* [69], *Euryale ferox* [70], and sweet potato starch [71] all exhibited significantly increased degrees of gelatinization. This transition from ordered to disordered structure facilitates enzymatic hydrolysis but may also lower gelatinization temperature and produce excessive viscosity during reconstitution, thereby introducing potential sensory drawbacks.

High shear forces affect protein structure mainly by disrupting hydrogen bonds and disulfide bonds that stabilize higher-order conformations. Hydrogen bonds help maintain secondary structures such as α-helices and β-sheets, whereas disulfide bonds covalently link polypeptide chains, supporting tertiary and quaternary structures. When shear forces are applied, these non-covalent bonds may break, causing protein unfolding and exposure of hydrophobic groups, which in turn alters the protein’s physicochemical and functional properties, thereby significantly improving emulsion stability and in vitro digestibility in materials such as ginseng powder [72], astragalus membranaceus [73], gastrodia elata [74], and *Acheta domesticus* powder [75]. Zhao et al. [58] studied soy protein isolate (SPI) powders prepared with different ultrafine grinding times (0–8 h). Ultrafine grinding improved the solubility, foaming, and emulsifying properties of SPI. In terms of secondary structure, α-helix, β-sheet, and random coil contents increased, while β-turn content decreased. Grinding time also affected the volatile flavor compounds of SPI, indicating that appropriate grinding can modulate both functional and flavor properties.

## 4. Applications of Superfine Grinding in the Processing of Edible Powders

The application value of superfine grinding in edible-powder processing can be best understood through representative scenarios involving plant by-products, whole grains and legumes, spices, and health-oriented functional ingredients.

### 4.1. Valorization of Plant-Based By-Products

Large quantities of by-products generated during fruit and vegetable processing—such as grape skins [76], apple pomace [77], and citrus peels [78]—are rich in polyphenols and dietary fiber, yet their highly compact lignocellulosic framework results in coarse mouthfeel and low gastrointestinal bioaccessibility, causing a persistent imbalance between high output and low valorization. Superfine grinding disrupts the cell-wall barrier through intense mechanical shear and thereby reconstitutes these low-value residues into high-activity functional ingredients. X-ray photoelectron spectroscopy analysis of psyllium husk powder showed that micronization not only altered monosaccharide composition but also significantly increased exposure of characteristic surface functional groups such as C-C/C-H while preserving the structural integrity of β-glycosidic linkages [79]. In grape skin residue [80], superfine processing partially degraded insoluble fiber and increased the proportion of soluble dietary fiber to 15.8%. When incorporated into bread or yogurt, the resulting fiber micropowder reinforces dietary fiber while mitigating the coarse gritty sensation typical of conventional bran. Likewise, after refinement, apple pomace [81] retained more than 90% of its total fiber, and the proportion of soluble fiber doubled. Such microstructural deconstruction not only reshapes hydration and rheology but also liberates a large amount of entrapped bioactives, thereby opening a practical route for high-value utilization of processing by-products.

### 4.2. Nutritional Functionalization of Whole Grains and Legumes

Whole grains and legumes, including soybean-based materials, retain structural components rich in fiber, B vitamins, and high-quality plant protein. However, their coarse mouthfeel and the chelating action of antinutritional factors such as phytic acid substantially reduce both sensory acceptability and true nutrient utilization. In this context, the key technological goal of superfine grinding is to reduce coarse particles to approximately 10–20 μm while preserving the full nutritional spectrum, thereby thoroughly disrupting fiber bundles and starch crystalline regions and effectively eliminating the rough bran-like sensation. Okara, a major by-product of soy processing, exemplifies this challenge [40]. Under conventional processing, its in vitro protein digestibility is very low. After superfine grinding of soybean matrices [82], not only is the granular mouthfeel markedly reduced, but endogenous protein molecules also undergo extensive conformational unfolding. The resulting improvement in hydration and solubility increases in vitro protein digestibility from 41.5% to 76.3%. Such restructuring of macromolecular rheology substantially broadens the application of soy protein in emulsion systems, protein powders, and plant-based meat analogs.

### 4.3. Fine Extraction and Preservation of Spice Flavor Compounds

The core quality of spices depends heavily on characteristic secondary metabolites, including alkaloids, oleoresins, and terpenoid essential oils [83]. Severe frictional heating during conventional ambient grinding readily causes volatilization of aroma-active compounds and oxidative deterioration of thermolabile substances, resulting in abrupt flavor loss in spice powders [84]. To reconcile superfine grinding with flavor preservation, cryogenic grinding using liquid nitrogen or other deep-cooling strategies—often maintaining the system at 25 °C or below—has become an essential option [85].

Under cryogenic protection, ginger micropowder [86] retains its intrinsic pungent flavor while exhibiting improved flowability and protein solubility. Saxena et al. [87] demonstrated that cryogenic micronization preserved the full-spectrum characteristic oleoresins and antioxidant activity of coriander powder (*Coriandrum sativum* L.). Ghodki et al. [88] further showed that the grinding temperature range from −120 °C to 40 °C strongly affected the quality of black pepper, and that under the extreme cryogenic condition of −120 °C the resulting micropowder retained high levels of volatile oil and mineral elements. At the parameter-optimization level, Goswami et al. [89] micronized cassia to 60 μm while achieving a high volatile-oil retention of 2.9 mL/100 g under a very low energy input of 31.3 kJ/kg, together with only minimal color deviation (ΔE* < 3). Singh et al. [90] further identified the temperature window from −110 °C to −50 °C as the safe range for preserving clove oil in cloves during cryogenic grinding; once the operating temperature approached 55–85 °C, flavor compounds underwent severe and irreversible losses.

### 4.4. Development of Health-Oriented Functional Ingredients

For high-value functional ingredients such as edible fungi and tea-derived materials, key efficacy is mainly associated with fungal polysaccharides, triterpenes, tea polyphenols, and related compounds. These macromolecules are often embedded deeply within rigid chitinous or lignified cell-wall frameworks, rendering conventional disruption methods inefficient and leading to substantial loss of target nutrients. Superfine mechanical force not only reduces particles physically but also increases the bioaccessibility of active molecules biochemically.

In fungal deep processing, Guo et al. [91] prepared *Sanghuangporus vaninii* superfine powder with a very narrow particle-size distribution. Intense cell-wall disruption directly promoted the release of intracellular polysaccharides, flavonoids, and triterpenoids into the solvent, and the solubility of polysaccharides also increased significantly. Ming et al. [48] compared the micronization kinetics of shiitake mushroom caps and stems and found that, at an average particle size of approximately 0.5 μm, grinding not only reduced the angle of repose and angle of slide, indicating improved flowability, but also generated a loose and porous microstructure that substantially increased the water solubility index and thermodynamic stability. In tea ingredient development, Hu et al. [92] reported that after superfine grinding of green tea powder, the exposure of polyphenols and catechins at the particle surface decreased whereas the content of water-soluble carbohydrates increased, thereby suppressing bitterness while preserving freshness and enhancing the antioxidant capacity of the overall extract. Tu et al. [93] further found that 600-mesh superfine black tea powder retained higher levels of tea polyphenols and theaflavins, significantly inhibited pancreatic lipase activity, and showed marked physiological benefits in improving lipid metabolism in animal models. Collectively, these findings indicate that superfine grinding can effectively overcome the sedimentation tendency and flavor heterogeneity associated with conventional tea powders.

### 4.5. Integrated Evaluation of Application Scenarios

Across the four representative application scenarios discussed above, two general industrial patterns can be identified. First, the economic return of superfine grinding depends strongly on end products with high added value and pronounced functional differentiation. Its advantages are especially evident in medicinal-and-edible resources, such as fungal polysaccharides and tea polyphenols, and in spices requiring precise retention of characteristic compounds, whereas the high costs associated with equipment depreciation and grinding energy consumption make the technology difficult to justify for bulk low-value raw materials. Second, all application scenarios face the same fundamental limitation: a single grinding technology cannot solve all quality-related problems simultaneously. Batch-to-batch variability and potential heavy-metal exposure in plant by-products, enzymatic lipid rancidity in whole grains, and the thermodynamic volatility dilemma of spices all demonstrate that physical cell-wall disruption alone is insufficient to ensure high-quality preservation. This industrial reality directly motivates the multi-technology strategies discussed in Section 6, including microencapsulation, resistance-reduction pretreatment, and molecular co-processing.

## 5. Limitations and Technical Challenges

The foregoing analysis indicates that superfine grinding has substantial engineering value in modulating the physicochemical properties and functionality of powders. However, when a single mechanical micronization technology is pushed toward extreme refinement, it inevitably gives rise to physicochemical and engineering limitations that cannot be eliminated merely by adjusting process parameters. These issues constitute the major barriers to the transition of superfine grinding toward more advanced industrialization and standardization.

### 5.1. Absence of Unified Evaluation Standards

At present, no unified evaluation standard or testing specification has been established domestically or internationally for food-grade superfine powders. Serious discrepancies therefore exist in the definition of key quality indicators and their judgment thresholds, constituting the most significant methodological obstacle to the standardized application of this technology. In particle-size classification, for example, no widely accepted threshold has been established for defining a superfine powder. Some studies use a median diameter of D50 ≤ 20 μm as the principal criterion, whereas others adopt a cumulative particle-size criterion of D90 ≤ 40 μm. This inconsistency means that products labeled as “superfine powders” may differ by several-fold in actual physical particle size, which directly undermines the comparability of functional data across studies and reduces the scientific reliability of the existing evidence base.

### 5.2. Physical-Stability Dilemma: Spontaneous Agglomeration Driven by Elevated Surface Energy

When particles are ground to extremely small sizes, the exponential increase in specific surface area gives rise to a series of physicochemical stability problems that are difficult to reverse. From a thermodynamic perspective, the increase in specific surface area markedly elevates the surface free energy of particles. To reduce this high-energy state, particles readily undergo spontaneous aggregation through van der Waals interactions and hydrogen bonding, thereby forming dense secondary agglomerates. Such spontaneous aggregation not only offsets the gain in specific surface area achieved by superfine grinding but also sharply increases hygroscopicity, making powders prone to moisture uptake and caking during storage. As a result, flowability deteriorates and the difficulty of mixing and conveying in industrial operations increases substantially [42,94].

In terms of chemical stability, the exposure of highly active surfaces increases the opportunity for functional groups to contact oxygen, thereby markedly accelerating the oxidation of labile components such as unsaturated fatty acids and directly shortening product shelf life. In addition, localized heating generated by mechanical friction can degrade thermolabile substances including vitamins, volatile essential oils, and polyphenols, whereas the introduction of low-temperature control systems entails high operation and maintenance costs.

### 5.3. Deterioration of Sensory Quality

Although excessive superfine grinding can enhance nutrient accessibility, it can also cause irreversible deterioration in sensory quality, particularly in plant by-products, whole grains, and starchy materials. From the perspective of mouthfeel and texture, micronized particles tend to agglomerate and are difficult to redisperse. When incorporated into liquid or semisolid food matrices, they may therefore generate pronounced grittiness and roughness, seriously compromising the original smooth texture of the product. More importantly, when starchy materials are subjected to excessive mechanical impact, their internal crystalline structure becomes disrupted and disordered, resulting in abnormal gelatinization behavior during reconstitution and heating. This can readily produce thick, highly viscous, dough-like pastes. Likewise, excessive refinement of plant tissues rich in pectin and dietary fiber readily promotes the formation of highly viscous gel networks upon hydration. Such sticky gel-like textures not only mask the authentic flavor of the food but also impede the extension of these powders into premium ready-to-drink or snack-food applications.

### 5.4. Energy-Consumption and Cost Barriers

The fundamental mechanics of superfine grinding determine its high energy demand and cost burden. First, the process imposes stringent requirements on equipment hardness, wear resistance, and precision control, including temperature regulation, gas-flow control, and dust removal. Core comminution components often rely on expensive materials such as silicon carbide or diamond, thereby creating a high entry threshold for small and medium-sized enterprises in both installation and maintenance. More fundamentally, as particles become increasingly fine, the pre-existing microcracks and structural defects within the powder decrease, while the resistance of the particles to tensile and compressive fracture increases sharply, often in an exponential manner [95]. Under such conditions, the large amount of externally supplied mechanical energy can no longer be efficiently converted into surface fracture energy; instead, most of it is dissipated ineffectively as frictional heat and acoustic energy [96]. Attempts to overcome this grinding limit by prolonging residence time further intensify wear of the grinding media and chamber, ultimately trapping the process in an industrial deadlock characterized by high energy consumption, low output, and severe equipment depreciation.

### 5.5. Safety Risks and Regulatory Concerns

The production and ingestion of extremely fine particles are accompanied by multiple safety concerns that cannot be overlooked. During manufacturing, prolonged severe friction may cause spalling of alloy wear-resistant parts, thereby increasing the baseline risk of excessive heavy-metal contamination in powders. Meanwhile, high concentrations of superfine airborne dust in processing environments can promote adsorptive microbial contamination and also constitute a serious dust-explosion hazard. From the perspective of physiological toxicology, accidental generation of nanoscale particles (<100 nm) during excessive refinement raises additional concerns. Some studies have suggested that ultrafine particles, particularly those at the nanoscale, may exhibit enhanced penetration behavior and could potentially interact with biological barriers; however, the in vivo behavior and long-term health implications of food-derived nano/submicron particles in humans remain insufficiently characterized [97]. Current evidence is largely based on in vitro and animal-model studies, and direct extrapolation to dietary exposure scenarios should be made with caution; the limited availability of in vivo safety data represents an important consideration for the large-scale application of this technology. Further systematic toxicological and clinical investigations are therefore warranted before definitive conclusions can be drawn regarding the safety of extremely fine food particles.

## 6. Multi-Process Synergy: Modification Strategies for Overcoming Single-Technology Bottlenecks

As discussed above, reliance on superfine grinding alone often leads to spontaneous particle agglomeration, poor water solubility, loss of bioactivity, and sharply increasing energy consumption, all of which restrict its broader application in the food industry. To overcome these bottlenecks fundamentally, cross-technology synergy has become a frontier topic in food powder engineering. By integrating superfine grinding with biological degradation, thermal or physical pretreatment, surface microencapsulation, and molecular assembly, it becomes possible to comprehensively remodel the dispersion stability, flowability, and targeted-delivery behavior of powders. Current mainstream strategies include biological resistance-reduction approaches such as enzymatic hydrolysis and fermentation; multi-physical-field pretreatments such as ultrasound-microwave coupling, extrusion, and steam explosion; wet interfacial modification through emulsification–homogenization and microencapsulation; phase-state reconstruction via spray and spray–freeze drying; and active molecular empowerment through mechanochemical co-processing and electrospraying. For its mainstream multi-technology integration strategies, please see Table 2 below.

### 6.1. Front-End Pretreatment and Substrate Resistance-Reduction Mechanisms

#### 6.1.1. Enzymatic Hydrolysis and Fermentation

**Figure 5:**
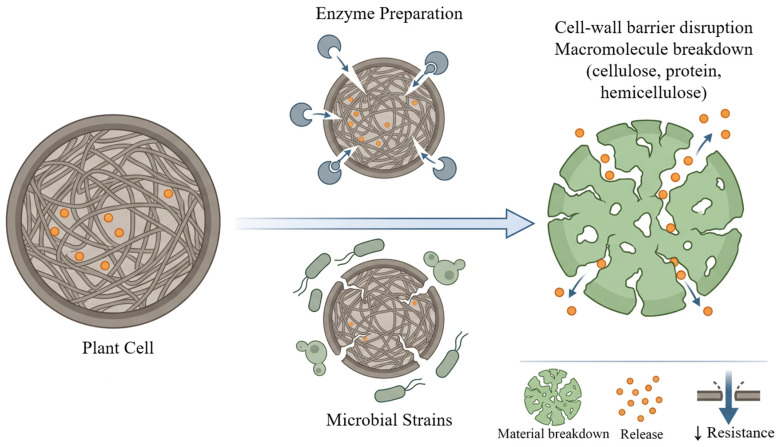
Schematic principle of enzymatic hydrolysis and fermentation pretreatment.

For agro-industrial by-products such as grape seed meal and wheat bran, which are characterized by highly compact cell walls, extensive polymer crosslinking, and severe entrapment of endogenous nutrients, biological pretreatment can precisely soften the structure and reduce grinding resistance. Mechanistically, food-grade enzyme systems such as cellulase, hemicellulase, and xylanase, or specific fermentation strains such as *Lactobacillus plantarum* and *Saccharomyces cerevisiae*, selectively cleave the crosslinked lignocellulosic network at the molecular scale, thereby weakening the Young’s modulus and mechanical toughness of the substrate, as illustrated in Figure 5. This not only greatly reduces fracture-energy consumption during subsequent grinding but also promotes deep release of bound polyphenols and flavonoids. Li et al. [98] used high-speed homogenization coupled with enzymatic treatment to modify okara dietary fiber and significantly improved its modulation of wheat-starch pasting dynamics, while reinforcing the internal hydrogen-bond network and thereby enhancing freeze–thaw stability and water-holding capacity of the gel system. Zhang et al. [99] demonstrated that cascade treatment combining superfine grinding with cellulase and feruloyl esterase effectively destroyed the microstructural barrier of bran, increasing free phenolic acid release from 59.94 μg/g to 329.14 μg/g and substantially enhancing α-glucosidase inhibitory potential in baked matrices. Yang et al. [100] further showed that combined ball milling and compound enzymatic hydrolysis of Japanese grape pomace disrupted the crystalline network of insoluble fiber and produced materials with pronounced lipid-lowering and glucose–lipid metabolic regulatory effects in mouse experiments.

#### 6.1.2. Extrusion and Steam Explosion Pretreatment

**Figure 6:**
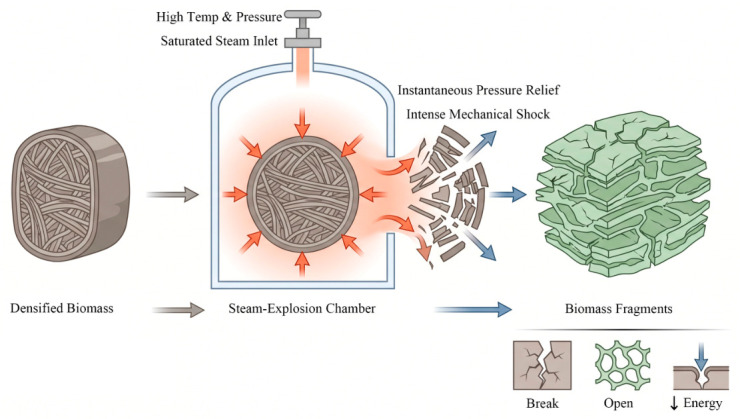
Schematic principle of steam-explosion pretreatment.

Extrusion and steam explosion constitute highly efficient nonchemical pretreatment routes for reducing comminution resistance. Extrusion applies intense screw-induced shear to forcibly strip biomass frameworks, whereas steam explosion uses penetration of high-temperature, high-pressure saturated steam followed by millisecond-scale pressure release to generate adiabatic expansion and flash-evaporation cavitation, thereby fundamentally destroying dense crystalline regions in cellulose (Figure 6) [101]. This explosive porous restructuring not only sharply reduces the mechanical-energy demand of subsequent superfine grinding but also geometrically suppresses the thermodynamic tendency of ultrafine particles to reagglomerate because of excessive surface energy [102]. Wang et al. [103] found that steam-explosion pretreatment of wheat bran increased extrusion efficiency by 36%; after equivalent grinding, cell-wall fragmentation was more complete and the equivalent content of soluble dietary fiber increased to 241.75%. Kong et al. [104] developed a coupled process combining steam explosion with superfine grinding, which increased the yield of superfine wheat-bran powder by 49.09% while keeping the median particle size below 75 μm. More importantly, powders produced by this coupled process outperformed those prepared by mechanical grinding alone in oil-holding capacity, bile-salt binding, and DPPH scavenging activity (87.68%).

#### 6.1.3. Ultrasound–Microwave Synergy

**Figure 7:**
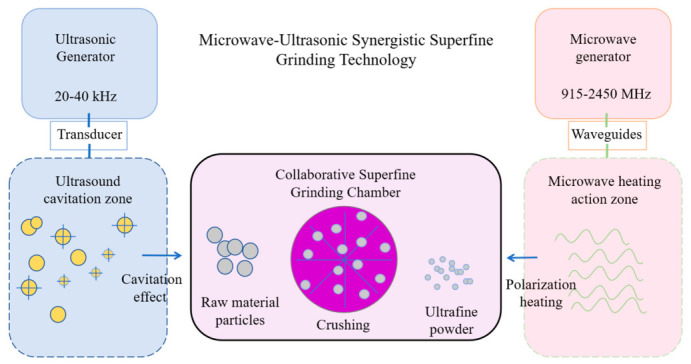
Schematic principle of ultrasound–microwave synergy.

The introduction of coupled ultrasonic and microwave fields opens a new route for noncontact optimization of grinding processes [105]. Cavitation bubbles generated by high-intensity ultrasound in liquid systems release microjets and shock waves upon transient collapse, causing multidirectional disruption of particle surfaces and thereby markedly reducing grinding resistance. Microwave dielectric polarization, in turn, provides volumetric heating, which helps avoid localized hot spots associated with conventional mechanical friction and thus better preserves the spatial conformation of thermolabile molecules (Figure 7) [106,107]. Jiang et al. [108] used ultrasound–microwave synergy to modify insoluble dietary fiber from corn bran and successfully generated a loose and porous surface morphology. Along with reduced crystallinity, this treatment substantially improved both bile-salt adsorption efficiency and in vitro hypoglycemic activity.

### 6.2. Wet-State Restructuring and Stabilization of Microstructure

#### 6.2.1. Wet Emulsification and Homogenization

The combined use of wet emulsification and homogenization represents a colloid–chemical solution to the thermodynamic aggregation and poor hydration compatibility of superfine powders. In this route, solid powders are first refined to the submicron scale and then combined with amphiphilic macromolecules such as phospholipids or whey proteins. Under the high-energy shear field of microfluidization or high-pressure homogenization, emulsifier molecules rapidly adsorb and self-assemble at the solid–liquid interface between powder particles and the continuous phase, thereby forming a dense interfacial protective film [109]. This steric and electrostatic barrier not only prevents secondary agglomeration but also enables excellent compatibility between strongly hydrophobic bioactives and aqueous systems. The strategy is therefore particularly useful for processing oil-rich fruit and vegetable powders and protein–polysaccharide composite microparticles.

#### 6.2.2. Spray Drying and Spray–Freeze Drying

**Figure 8:**
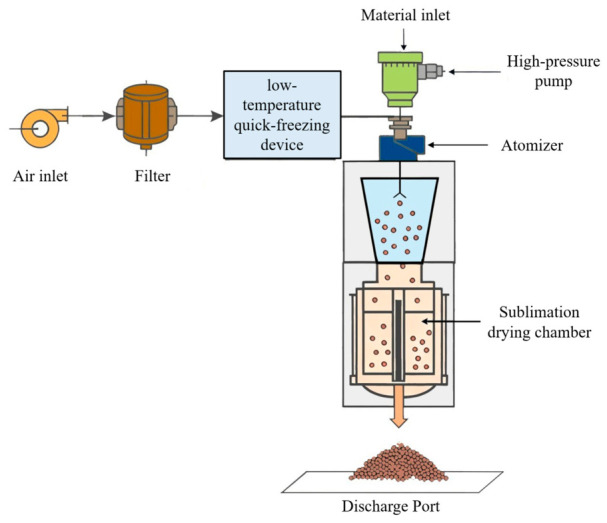
Schematic principle of spray–freeze drying.

Spray drying and spray–freeze drying are particularly suitable for preparing high-value edible superfine powders derived from materials such as goji berry extract and fruit or vegetable juice concentrates [110]. Atomization in the spraying step transforms the liquid feed into fine droplets with greatly enlarged specific surface area. In spray–freeze drying (SFD), the combination of ultrafast freezing and subsequent vacuum sublimation of ice crystals yields an extremely porous sponge-like microstructure (Figure 8). This architecture improves flowability and reconstitution performance, mitigates the moisture-induced caking commonly observed in powders produced solely by superfine grinding, and maximizes retention of thermolabile bioactives by minimizing heat exposure during drying [111].

### 6.3. Surface Remodeling and Molecular Protection

#### 6.3.1. Microencapsulation

**Figure 9:**
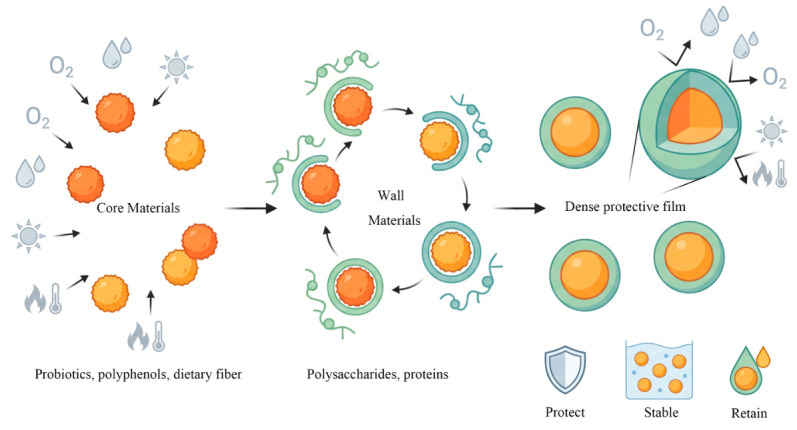
Mechanism of microencapsulation.

Microencapsulation uses food-grade polymeric wall materials, including polysaccharides and proteins, to physically enclose superfine powder cores within a three-dimensional protective matrix [112]. In essence, this approach creates an effective barrier against light, oxygen radicals, and moisture (Figure 9), making it a multifunctional solution to the major shortcomings of superfine powders, namely oxidation susceptibility, hygroscopicity, volatility, and coarse oral perception [113]. The strategy has already formed a relatively mature technological route in probiotic delivery [114] and polyphenol protection systems. For example, Gomes et al. [115] used inulin and OSA-modified starch as a dual-layer wall system to encapsulate superfine extract from Brazil nut cake. During 120 days of storage, seven phenolic compounds and five flavonoids were effectively protected from oxidative degradation, while the antioxidant capacity and selenium retention of the final product remained highly stable.

#### 6.3.2. Polyelectrolyte Dispersant Intervention

Polyelectrolyte dispersants such as sodium polymethacrylate act as strong electrostatic stabilizers because the main chain carries a high density of anionic carboxylate groups (-COO-), which can anchor effectively onto superfine particle surfaces. Through the combined action of electrostatic repulsion and steric hindrance of polymer chains, these additives can strongly suppress spontaneous aggregation driven by van der Waals attraction and thus improve grinding efficiency. However, because the regulatory compliance of such additives under food-additive legislation remains subject to strict scrutiny, their application in food-grade edible powders is currently far less common than in pharmaceutical formulations such as solid dispersions [116]. Any food-related use must therefore be evaluated carefully from both safety and regulatory perspectives.

### 6.4. Molecular Empowerment and Targeted-Delivery Networks

#### 6.4.1. Mechanochemical Co-Processing

Mechanochemical co-processing goes beyond simple physical premixing and can be regarded as an active, mechanically driven molecular reconstruction process. Under the intense mechanical field generated by high-energy grinding, for example in high-energy ball milling or opposed-jet pulverization, activated substrate particles acquire highly reactive surfaces and interact strongly with deliberately introduced functional additives such as proteins or cyclodextrins. Simultaneously, the crystal lattice of the particles may become distorted and even chemical bonds may be cleaved, allowing the system to form new supramolecular complexes through hydrogen bonding, electrostatic attraction, and other noncovalent interactions [117]. This solvent-free and environmentally benign route not only produces more uniform particle sizes but, more importantly, substantially improves the aqueous dispersibility and bioactivity of otherwise poorly water-soluble polyphenols and hydrophobic fibers. It is therefore emerging as a frontier strategy for ultrahigh-value modification of grain-processing by-products.

#### 6.4.2. Electrospraying

**Figure 10:**
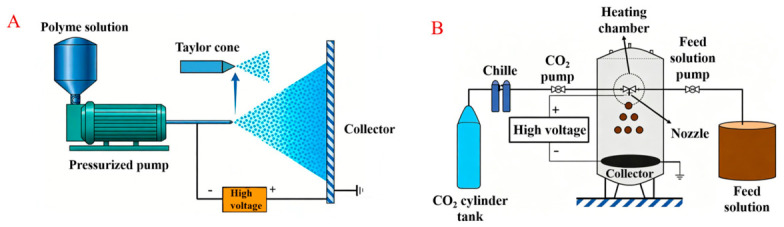
Schematic principles of electrospraying (**A**) and supercritical CO_2_-assisted microparticle preparation (**B**).

In electrospraying, a solution containing superfine active particles and wall materials such as sodium alginate or chitosan is exposed to a high-voltage electrostatic field (Figure 10). Charged droplets overcome surface tension under the polarized field to form a Taylor cone and then rapidly solidify in space into microscale or nanoscale core–shell carriers with highly uniform particle size [118]. In practical processing, this technique can also be combined with supercritical CO_2_-assisted particle formation. Electrospraying avoids destructive high temperatures and, through core–shell encapsulation efficiencies often exceeding 90%, enables precise protection of probiotics, polyphenols, unsaturated fatty acids, flavor compounds, and other functional factors from moisture, heat, oxidation, and mechanical stress [119]. Owing to its high storage stability, the approach shows considerable promise for developing long-shelf-life, highly stable fruit and vegetable ingredient systems.

#### 6.4.3. Multiscale Compounding

Based on spatial filling and interfacial synergy, multiscale compounding refers to the gradient blending of food powders with different particle sizes or categories after superfine grinding. Fine particles fill the voids among coarse particles, while particles of different scales establish physical barriers against each other. In this way, the common defects of single-scale superfine powders—such as agglomeration, poor flowability, caking during reconstitution, and insufficient solubility—can be effectively alleviated. Lin et al. [105] incorporated Qingke flour into wheat flour for bread making and found that the complete starch degradation rate of the dough decreased sharply from 80% to 41%, while the flavor profile of the baked product also changed substantially. These results provide an important basis for texture regulation in low-glycemic-index, nutritionally complete meal-replacement powders.

### 6.5. Other Modification Processes

Additional modification strategies have also been explored. Irradiation can induce chain scission of macromolecules, including polysaccharides and intracellular proteins, through high-energy physical bombardment and free-radical-mediated reactions, thereby facilitating destruction of dense cell-wall structures. Cheng et al. [120] showed that irradiation combined with superfine grinding of pine pollen accelerated release of superoxide dismutase and reconstructed migration pathways of anthocyanins and endogenous fatty acids at the microscale. Chemical graft modification, by contrast, exploits the large surface energy generated by superfine grinding as a reaction platform. Through carboxymethylation (-CH_2_COOH) or acetylation (-COCH_3_), free hydroxyl groups on cellulose chains can be replaced, fundamentally altering the hydrophilic–hydrophobic balance of the fiber surface. Xu et al. [121] demonstrated that superfine grinding combined with dual-enzyme treatment and subsequent carboxymethyl grafting of oil palm kernel expeller fiber markedly improved both viscosity modulation and inhibition of intestinal glucose diffusion.

**Table 2:** Mainstream multi-technology integration strategies.

Combined Strategy	Core Mechanism	Main Improvement	Applicable Matrices	Reference
Mechanochemical co-processing	Superfine grinding combined with high-energy milling and functional matrices to enable molecular-level recombination via interfacial hydrogen bonding, covalent coupling, and lattice distortion.	Breaks the particle-size limit and markedly improves hydration and rheological performance.	Bran, okara, cereals	[117]
Ultrasound–microwave coupling	Ultrasonic cavitation microjets tear cell walls, while microwave dielectric heating provides volumetric heating and avoids local thermal degradation.	Greatly enhances release and aqueous solubility of flavonoids, polysaccharides, and other actives.	Pueraria, medicinal-and-edible matrices, fruits and vegetables	[107]
Wet emulsification/homogenization	Superfine slurries combined with food-grade amphiphilic emulsifiers undergo interfacial restructuring under high-pressure shear, followed by dehydration into powder.	Produces highly stable composite micropowders with excellent dispersion and minimal secondary agglomeration.	Oil-rich systems, protein-containing matrices, fruit/vegetable concentrates	[109]
Spray drying/spray–freeze drying	Atomization expands specific surface area, and rapid cooling plus sublimation creates a highly porous structure.	Improves probiotic survival, storage stability, and bioaccessibility of thermosensitive compounds.	Probiotics, curcumin, anthocyanins and other heat-sensitive materials	[111]
Enzymatic hydrolysis/fermentation	Specific enzymes or strains selectively cleave crosslinked fiber or protein networks, reducing matrix rigidity at the source.	Releases encapsulated soluble dietary fiber, improves digestibility, and mitigates coarse mouthfeel.	Wheat bran, soybean meal, wheat germ and other hard matrices	[98]
Extrusion/steam explosion	High-temperature high-pressure steam release or screw extrusion destroys fibrous frameworks and crystalline domains before grinding.	Rebuilds surface area, improves solubility, and reduces mechanical-energy demand.	Bran, whole grains, seed husks and other hard biomasses	[102]
Electrospraying	High-voltage electrostatic atomization solidifies droplets into core–shell micro/nanocarriers.	Enables low-thermal-loss encapsulation and strong storage and controlled-release performance.	Free polyphenols, volatile flavor peptides	[118]
Multiscale compounding	Powders of different sizes are blended to exploit void filling and interfacial synergy.	Improves flowability, reduces agglomeration, and optimizes reconstitution and nutritional performance.	Meal-replacement systems, composite cereal matrices	[105]
Microencapsulation	Food-grade polymers form a dense protective layer around superfine powder particles.	Blocks oxygen/light/water damage, suppresses volatilization, masks off-flavors, and improves reconstitution.	Highly oxidizable, hygroscopic, or volatile superfine powders	[112]
Polyelectrolyte dispersants	Exogenous anionic polymers anchor to particle surfaces to generate electrostatic repulsion and steric hindrance.	Suppresses thermodynamic agglomeration and improves suspension-dispersion kinetics.	Dietary-fiber colloids, polymerized protein systems	[116]

## 7. Conclusions and Perspectives

### 7.1. Conclusions

This review systematically examined the mechanisms, physicochemical effects, and multi-technology synergy strategies of superfine grinding in the preparation of edible powders. Based on the available evidence, the following conclusions can be drawn.

(1) The physicochemical regulation exerted by superfine grinding is strongly matrix-specific. By effectively disrupting cell walls and tissue architecture, the mechanical force field markedly reduces particle size and enlarges specific surface area. This physical process not only improves hydration kinetics and dispersion behavior but also promotes the release of endogenous active constituents such as polyphenols and polysaccharides, thereby enhancing the in vitro bioaccessibility of relevant nutrients. Different raw materials, including fiber-rich, starch-rich, and lipid-rich matrices, exhibit systematic differences in their response to specific mechanical force fields such as impact, shear, and friction.

(2) The quality-improving effect of any single grinding technology is constrained by physicochemical thresholds. Excessive refinement readily increases surface free energy and thereby induces spontaneous particle agglomeration, offsetting the benefits of micronization. In parallel, frictional heating promotes degradation of thermolabile compounds, lipid oxidation, and deterioration of the rheological behavior of macromolecules such as starch and pectin. Optimization of process parameters alone is therefore insufficient to balance the contradiction between extreme size reduction and the high retention of functional quality.

(3) Multi-technology synergy is an effective route for overcoming the limitations of single grinding technologies. The integrated combination of superfine grinding with biological resistance-reduction approaches, such as enzymatic hydrolysis and fermentation, physical pretreatments such as steam explosion, and post-processing strategies such as wet emulsification and microencapsulation, can effectively reduce energy consumption, suppress particle agglomeration, and protect functional ingredients through spatial isolation. Such integration represents a promising engineering direction toward food ingredients that combine high stability with strong functional activity.

### 7.2. Perspectives

Despite the considerable application potential of superfine grinding in food ingredient processing, major challenges remain in both fundamental mechanistic understanding and industrial translation. Future research should focus on the following directions.

(1) Establish standardized evaluation systems based on raw-material characteristics. Unified particle-size grading criteria and physicochemical testing specifications should be developed for different food matrices, such as whole grains, fruit and vegetable by-products, and medicinal-and-edible materials, to provide a reliable methodological basis for cross-study and cross-laboratory comparison and to support the formulation of sectoral and national standards.

(2) Deepen mechanistic research on food mechanochemistry. Future work should investigate, at the molecular level, how extreme mechanical stress induces noncovalent and covalent interactions among food macromolecules such as proteins, dietary fiber, and polyphenols. Quantitative evaluation of the role of mechanochemistry in improving water solubility and inducing macromolecular reorganization will provide theoretical support for its application in green, solvent-free food modification.

(3) Promote systematic optimization and life-cycle assessment (LCA) of multi-technology integrated processes. Most combined strategies remain at laboratory scale. Future efforts should therefore bridge the path from mechanistic validation to pilot-scale translation, systematically optimize multistage process parameters, and introduce LCA models to quantify overall energy consumption, carbon footprint, and economic feasibility, thereby advancing green and continuous production.

(4) Improve the safety-evaluation framework for submicron and nanoscale food particles. Potential exposure risks associated with extreme particle miniaturization should be taken seriously. Systematic in vivo toxicological studies are needed to clarify transport kinetics, target-organ accumulation, and interactions with gut microbiota of superfine particles in the human gastrointestinal tract. Additional clinical and toxicological evidence would help to address current gaps in safety evaluation and regulatory governance of extremely fine food particles.

(5) Targeted technology–product matching recommendations for industrial application. Based on the integrated analysis in this review, the following technology recommendations can be proposed for representative edible-powder product categories:

Heat-sensitive spices and aromatic herbs (e.g., black pepper, clove, ginger, mint): cryogenic jet milling or cryogenic vibration milling (operating temperature ≤ −10 °C) coupled with microencapsulation to preserve volatile oils and oleoresins.

Whole grains, legumes, and bran-rich by-products (e.g., wheat bran, okara, oat bran): steam explosion + superfine grinding or enzymatic hydrolysis + ball milling to enhance soluble dietary fiber content and reduce gritty mouthfeel.

Polyphenol- and pigment-rich fruit/vegetable by-products (e.g., grape pomace, apple pomace, hawthorn): wet ball milling or colloid milling combined with spray–freeze drying to maximize anthocyanin and polyphenol retention.

Functional medicinal-and-edible matrices (e.g., *Ganoderma lucidum* spore powder, *Lycium ruthenicum* Murray, ginseng): high-pressure homogenization or low-temperature wet grinding coupled with electrospraying for controlled-release delivery of polysaccharides and triterpenes.

Probiotic and high-value functional ingredient powders: spray–freeze drying combined with double-layer microencapsulation to ensure storage stability and gastrointestinal viability.

## Data Availability

No new data were created or analyzed in this study. Data sharing is not applicable to this article.
